# The clathrin-binding motif and the J-domain of *Drosophila Auxilin *are essential for facilitating Notch ligand endocytosis

**DOI:** 10.1186/1471-213X-8-50

**Published:** 2008-05-08

**Authors:** Vasundhara Kandachar, Ting Bai, Henry C Chang

**Affiliations:** 1 Department of Biological Sciences, Purdue University, 915 West State Street, West Lafayette, Indiana 47907-2054, USA

## Abstract

**Background:**

Ligand endocytosis plays a critical role in regulating the activity of the Notch pathway. The *Drosophila *homolog of auxilin (*dAux*), a J-domain-containing protein best known for its role in the disassembly of clathrin coats from clathrin-coated vesicles, has recently been implicated in Notch signaling, although its exact mechanism remains poorly understood.

**Results:**

To understand the role of auxilin in Notch ligand endocytosis, we have analyzed several point mutations affecting specific domains of dAux. In agreement with previous work, analysis using these stronger *dAux *alleles shows that dAux is required for several Notch-dependent processes, and its function during Notch signaling is required in the signaling cells. In support of the genetic evidences, the level of Delta appears elevated in *dAux *deficient cells, suggesting that the endocytosis of Notch ligand is disrupted. Deletion analysis shows that the clathrin-binding motif and the J-domain, when over-expressed, are sufficient for rescuing *dAux *phenotypes, implying that the recruitment of Hsc70 to clathrin is a critical role for dAux. However, surface labeling experiment shows that, in *dAux *mutant cells, Delta accumulates at the cell surface. In *dAux *mutant cells, clathrin appears to form large aggregates, although Delta is not enriched in these aberrant clathrin-positive structures.

**Conclusion:**

Our data suggest that *dAux *mutations inhibit Notch ligand internalization at an early step during clathrin-mediated endocytosis, before the disassembly of clathrin-coated vesicles. Further, the inhibition of ligand endocytosis in *dAux *mutant cells possibly occurs due to depletion of cytosolic pools of clathrin via the formation of clathrin aggregates. Together, our observations argue that ligand endocytosis is critical for Notch signaling and auxilin participates in Notch signaling by facilitating ligand internalization.

## Background

The Notch pathway, a highly conserved signaling module, participates in diverse aspects of animal development, including cell proliferation, differentiation, and pattern formation [1,2]. Upon ligand binding, Notch undergoes proteolytic processing, resulting in the release and the nuclear translocation of NICD (Notch intra cellular domain) for transcriptional activation [3]. As the Notch receptor and its ligands are widely expressed, the activities of this important pathway need to be regulated at multiple levels to prevent inappropriate signaling output. Recent evidence from several systems has suggested that ligand endocytosis plays a key role in regulating the activity of this cascade, although its exact function in Notch signaling remains unclear.

Genetic analysis using *Drosophila *has identified several factors required for ligand endocytosis during Notch signaling. In *Drosophila*, there are two known Notch ligands, Delta (Dl) and Serrate (Ser), both of which appear to utilize an ubiquitin-mediated endocytic pathway to activate Notch [4-8]. While polyubiquitination is known to facilitate protein degradation, covalent addition of a single ubiquitin moiety to the cytoplasmic tails of membrane proteins can serve as an internalization signal [9,10]. The ubiquitination of Dl and Ser for internalization is mediated by *neuralized (neur*) and *mind bomb (mib1)*, two structurally unrelated but functionally similar E3 ubiquitin ligases [4,7,11-16]. The subsequent recruitment of ubiquitinated Notch ligands into clathrin-coated vesicles is thought to depend on *liquid facets (lqf*, the *Drosophila *homolog of epsin), a cargo-specific clathrin adaptor [17-20]. Dynamin, a GTPase required for the pinching-off of clathrin-coated vesicles (CCVs) from the plasma membrane, is also required for this event [21,22]. Moreover, during the asymmetric cell division of sensory organ precursors, Rab11 (a GTPase associated with recycling endosomes) and Sec15 (a component of the exocyst complex) have been shown to promote Notch signaling and regulate Dl trafficking [23,24].

**Figure 1:**
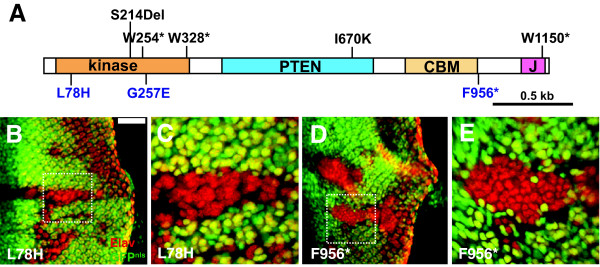
***dAux *mutations cause defects in photoreceptor specification**. (A) A schematic diagram of *Drosophila Auxilin*, consisting of a N-terminal kinase domain (orange), a PTEN-related domain (blue), clathrin binding motifs (CBM, yellow), and a J-domain (pink). The molecular lesions in eight *dAux *alleles are shown, and those isolated with a FRT chromosome are indicated in blue. (B-E) Fluorescent micrographs of larval eye discs from (B, C) *Act5C>FLP/+; FRT*^5-5*Z*3515^, *dAux*^*L*78*H*^*/FRT*^5-5*Z*3515^, *ubi-GFP*^*nls *^and (D, E) *Act5C>FLP/+; FRT*^5-5*Z*3515^, *dAux*^*F*956^*/FRT^5-5*Z*3515^, *ubi-GFP*^*nls *^animals. The mosaic discs are stained for Elav (red), which labels the nuclei of neuronal cells, and mutant tissues are marked by the absence of nuclear-GFP expression (green). (C, E) Boxed areas of *dAux*^*L*78*H *^and *dAux*^*F*956^* clones from B and D are shown at a higher magnification. In all the panels, anterior is to the left. Scale bar, 50 μm.

Mutations in *Drosophila auxilin *(*dAux*), a J-domain-containing regulator in clathrin-mediated transport, were recently shown to disrupt Notch-dependent processes [25-27]. Although originally identified as a factor promoting the assembly of clathrin cage from free triskelia [28], efforts investigating the role of auxilin during clathrin-mediated endocytosis (CME) have mostly focused on its cooperation with Hsc70 in mediating the disassembly of clathrin coats form nascent CCVs [29]. In mammals, there are two different auxilin-like molecules (auxilin 1 and GAK/auxilin 2), differing in their tissue distributions and the presence of a N-terminal Ark family kinase domain [29-31]. In contrast, the *Drosophila *genome contains only one auxilin ortholog, which is structurally more similar to GAK, as it contains a N-terminal kinase domain, followed by a PTEN (phosphatase and tensin) homologous region, a clathrin-binding domain, and a C-terminal dnaJ domain (Figure 1A) [32]. Biochemical data suggest that the CBM (clathrin-binding motif) and J-domain recruit ATP-bound Hsc70 to CCVs and stimulate the ATPase activity of Hsc70, which drive the disassembly of clathrin coats [30,33,34]. The PTEN-related region appears to be critical for the membrane recruitment of auxilin to clathrin-rich regions [35,36]. The N-terminal kinase is known to phosphorylate subunits of AP1 and AP2 adaptor complexes, possibly modulating the affinity of these adaptors for cargoes [31,37-40]. However, as not all auxilin homologs (for example, yeast swa-2 and C. elegans auxilin) contain the kinase and the PTEN-related region [41-43], the physiological relevance of these domains remains to be determined.

Initially, the identification of auxilin as a component of the *Drosophila *Notch pathway suggests that, to activate Notch, ligand endocytosis needs to proceed past the step of clathrin cage disassembly. This implies that the relevance of ligand endocytosis is not the internalization per se, but a transit of ligand through subsequent endocytic compartments. However, this interpretation hinges on the assumption that auxilin participates solely in clathrin uncoating under physiological conditions. Recent in vitro investigations have suggested several new functions of auxilin during CME [32], including facilitating clathrin exchange during CCV formation [44,45], binding to adaptor complexes [46], binding to dynamin to facilitate the constriction of coated pits [47], and chaperoning dissociated clathrin for subsequent rounds of endocytosis [48]. Thus, to understand the significance of ligand endocytosis in Notch, it is critical to determine whether dAux is exclusively involved in clathrin uncoating or has additional roles during CME under physiological conditions.

Using strong *dAux *alleles generated from an *FRT*-containing chromosome, we have obtained compelling evidences that *dAux *mutations cause defects in multiple Notch-dependent processes and *dAux *function is required in the signaling cells. Although the relevant cargo for dAux during Notch signaling seems to be Dl, the endocytic function of dAux is not limited to the Notch ligand, as the internalizations of other membrane proteins also appear disrupted. Deletion analysis shows that over-expressed CBM and J-domain are sufficient for *dAux *function, suggesting that the recruitment of Hsc70 to CCVs is a core function of dAux. Furthermore, while the disruption of *dAux *function alters the sub-cellular clathrin distribution, Dl appears to be trapped at the cell surface, away from the abnormal clathrin-positive structures. Together, our data support a model, in which dAux facilitates Notch ligand endocytosis by regulating clathrin dynamics. Moreover, the fact that Dl is trapped at the plasma membrane in *dAux *mutants suggests that the linking of *dAux *to the Notch pathway does not exclude the possibility that the Notch ligand internalization per se plays a pivotal role in activating Notch.

## Results

### Mutations in *dAux *kinase- or J-domains disrupt photoreceptor specification

To further understand the role of dAux under physiological conditions, we have isolated additional *dAux *mutations from several rounds of F_2 _non-complementation screens. We have nine mutations in *dAux*, and, for eight of these nine alleles, the molecular lesions are known (Figure 1A). Furthermore, three of these alleles, namely *dAux*^*L*78*H*^, *dAux*^*F*956^*, and *dAux*^*G*257*E*^, were generated from an *FRT*-containing chromosome; thus permitting *dAux *phenotypic analysis in clones.

To assess the strength of these *dAux *alleles, the lethal phases of animals carrying these *dAux *mutations over *Df(3R)ED5021*, a deletion (82A1-A2) that removes the entire *dAux *locus, were determined. Five of these alleles (*dAux*^*S*214^*, dAux^*W*254^*, *dAux*^*W*328^*, *dAux*^*F*956^*, and *dAux*^*W*1150^*), when trans-heterozygous with the deletion, died prior to the larval stage. Three of them, *dAux*^*S*214^*, *dAux*^*W*254^*, and *dAux*^*W*328^*, all contain nonsense mutations within the N-terminal kinase domain, suggesting that they are strong or null alleles. The early lethal phase of *dAux*^*F*956^* and *dAux*^*W*1150^*, two nonsense mutations near the C-terminus, most likely reflects the importance of the J-domain in *dAux *function. This analysis indicated that *dAux*^*L*78*H *^and *dAux*^*G*257*E*^, two missense mutations disrupting highly conserved residues in the sub-domain II and IX of the N-terminal kinase domain [49] respectively, are weaker alleles. Thus, the allele series, in descending allelic strength, can be described as: S214*≈W254*≈W328* > F956*≈W1150* > L78H≈G257E > I670K (*dAux*^*I*670*K *^is a viable hypomorph).

Previous studies have shown that mutations in the PTEN-related region cause defects in photoreceptor specification [25,26]. To test whether mutations in other dAux domains have similar effects, FLP-induced mutant clones of *dAux*^*L*78*H *^and *dAux*^*F*956^*, mutations disrupting the kinase and the J-domain respectively, were stained with α-Elav, which labels the nuclei of neuronal cells [50]. In wild-type tissues, an organized set of eight Elav-positive cells is formed in each cluster. In contrast, as shown in Figure 1B–E, increased numbers of disorganized Elav-positive cells were seen in both *dAux*^*L*78*H *^and *dAux*^*F*956^* mutant clones (Figure 1B–E, indicated by the absence of the nuclear GFP). This suggests that the integrity of the kinase and the J-domain, in addition to the PTEN-related region, is important for the function of dAux during neuronal cell specification. In addition, this neural hypertrophy exhibited by *dAux*^*F*956^* tissues seemed to be more severe than those of *dAux*^*L*78*H *^(in the sense that additional *dAux*^*L*78*H *^Elav-positive cells appeared to be grouped in clusters, whereas *dAux*^*F*956^* cells did not), correlating well with the allelic strength defined by the lethal phase analysis.

### Notch-dependent proneural enhancement and lateral inhibition are both disrupted in *dAux *mutant tissues

The *Drosophila *compound eye consists of regular arrays of ~800 ommatidia, each containing eight stereotypically positioned photoreceptors (R1-R8) and other accessory cells. The formation of this elaborate pattern is initiated in the larval eye imaginal disc along the morphogenetic furrow (MF, an indentation in the eye disc epithelium), which sweeps from posterior to anterior across the eye disc [51]. Behind the furrow, undifferentiated cells are sequentially recruited to adopt distinct developmental fates. To further understand the roles of *dAux *in photoreceptor cell differentiation, we investigated the effect of *dAux *mutations on the specification of R8, the founder cell within each cluster. The specification of R8 cells is known to require at least two Notch-dependent events: 1) proneural enhancement: an up-regulation of *atonal *(*ato*, a proneural gene) [52] expression in cells near the morphogenetic furrow to confer them the capability to adopt a neuronal fate, and 2) lateral inhibition: a subsequent restriction of the broad Ato expression to one single R8 cell per cluster behind the MF [53,54].

**Figure 2:**
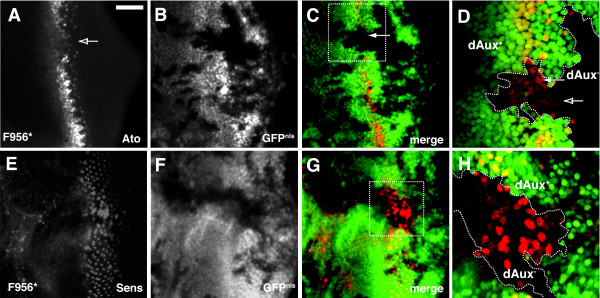
***dAux *mutations disrupt the proneural enhancement and the lateral inhibition during R8 specification**. Projected spinning disk confocal micrographs of *Act5C>FLP/+; FRT*^5-5*Z*3515^, *dAux*^*F*956^*/FRT^5-5*Z*3515^, *ubi-GFP*^*nls *^larval eye stained for Atonal (A-D, red) and Senseless (E-H, red). Regions marked with boxes in (C) and (G) are shown at a higher magnification in (D) and (H), respectively. *dAux*^*F*956^* mutant clones are indicated by the absence of nuclear GFP and outlined with dashed lines in (D) and (H). *dAux*^*F*956^* mutant cells with an elevated level of Atonal expression at the clone border are indicated by solid arrows (C and D). Furthermore, groups of Atonal-positive cells that fail to resolve into single Atonal-expressing cells are indicated by open arrows (A and D). Scale bar, 50 μm.

To determine if *dAux *is required for these processes, mosaic eye discs containing *dAux*^*F*956^* mutant clones were stained for Ato. As shown in Figure 2A, most *dAux*^*F*956^* mutant cells located near the MF expressed less Ato than their wild-type counterpart, suggesting that *dAux *has a role in the proneural enhancement event. Noticeably, some *dAux*^*F*956^* mutant cells at the clone border still expressed an elevated level of Ato (Figure 2C and 2D, solid arrows). One plausible explanation is that *dAux *functions non-cell-autonomously during Notch signaling (see below); therefore, the Notch pathway in these *dAux*^*F*956^* mutant cells could still be activated because they were juxtaposed to the wild-type cells.

Behind the MF in normal eye discs, the broad Ato expression is gradually restricted, by Notch-mediated lateral inhibition, to a single cell in each cluster. Unlike wild type, *dAux*^*F*956^* mutant tissues contained clumps of multiple Ato-positive cells that are not restricted into single Ato-expressing cells (Figure 2A, open arrows), suggesting that the process of restricting Ato expression was disrupted. To further confirm that lateral inhibition during R8 specification was affected by *dAux*, FLP-induced eye disc clones were stained for Senseless (Sens), a Zn finger-containing transcription factor expressed in the nuclei of R8 cells [55]. Unlike Ato, whose expression disappears in more mature R8 cells, *sens *expression persists in all R8 cells in the eye disc [55]. Similar to the Ato staining, clusters of extra Sens-positive nuclei were seen in *dAux*^*F*956^* mutant tissues after the furrow (Figure 2H). Together, these observations suggest that both proneural enhancement and lateral inhibition, two known Notch-dependent processes, require *dAux*.

### Mutations in *dAux *disrupt the formation of the dorsal-ventral wing boundary

To ask if *dAux *regulates other Notch-dependent processes, we examined the effect of *dAux *mutations on the formation of the dorsal-ventral (DV) boundary in the developing wing discs. In normal wing discs, cells in both the dorsal and ventral compartments express Notch. However, because of the modification of Notch receptor by *fringe *in the dorsal compartment [56,57], these dorsal cells respond preferentially to Dl signaling from cells in the ventral compartment. Conversely, the cells in the ventral half respond preferentially to the other Notch ligand, Ser, which is expressed in the dorsal half. As a result, margin-specific genes like *cut *are expressed in a stripe of cells along the DV border, in a Notch-dependent manner [58].

**Figure 3:**
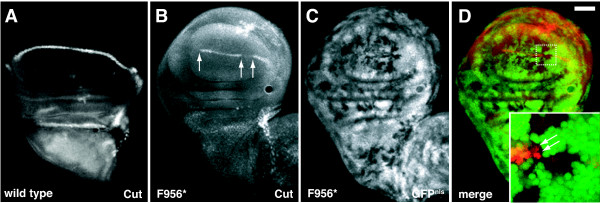
***dAux *mutations disrupt Cut expression at the DV boundary in developing wing discs**. Fluorescent micrographs of (A) wild-type and (B-D) *Act5C>FLP/+; FRT*^5-5*Z*3515^, *dAux*^*F*956^*/FRT^5-5*Z*3515^, *ubi-GFP*^*nls *^larval wing discs stained with 2B10 anti-Cut antibody (red). A mutant clone at the DV boundary (indicated by the white box) is shown at higher magnification in the inset in (D). *dAux*^*F*956^* mutant clones are indicated by the absence of nuclear GFP (C). Some *dAux*^*F*956^* cells (indicated by arrows) at the clone boundary are Cut-positive (see text for explanation). Scale bar, 50 μm.

To test if this process requires *dAux*, wild-type and *dAux*^*F*956^* mosaic wing discs were stained with 2B10 α-Cut antibody [59]. In wild-type wing discs, a stripe of Cut-positive cells was seen along the DV border (Figure 3A). In contrast, Cut staining was absent in *dAux*^*F*956^* mutant clones located at the DV border (Figure 3B–D), suggesting that *dAux *is required for the DV boundary formation in wing development. Again, at a higher magnification (Figure 3D, inset), some *dAux*^*F*956^* mutant cells at the clone border still expressed Cut, supporting the possibility that *dAux *functions non-cell-autonomously during Notch signaling (see below).

### dAux acts non-cell-autonomously during Notch signaling

**Figure 4:**
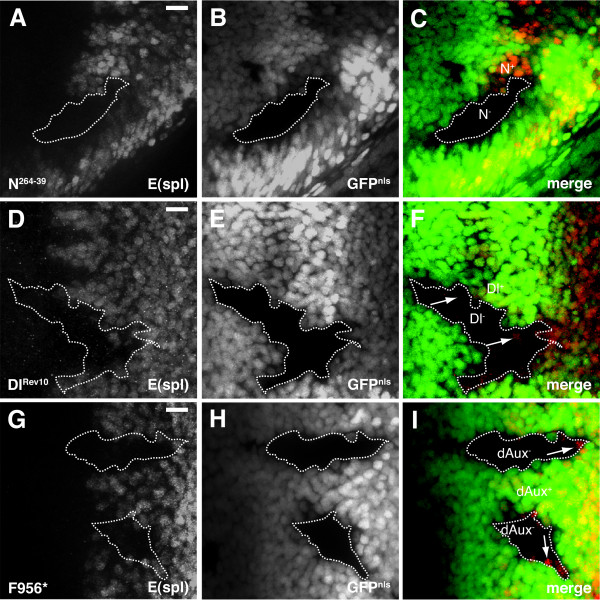
**dAux functions in the signal-sending cells during Notch signaling**. Projected spinning disk confocal images of (A-C) *N*^264-39^, *FRT*^9-2^*/ubi-GFP*^*nls*^, *FRT*^9-2^*; Act5C>FLP/+*, (D-F) *ey-FLP/+; FRT82B, Dl*^*RevF*10^*/FRT82B, ubi-GFP*^*nls *^and (G-I) *Act5C>FLP/+; FRT*^5-5*Z*3515^, *dAux*^*F*956^*/FRT^5-5*Z*3515^, *ubi-GFP*^*nls *^larval eye discs stained with mAB323 anti-E(spl) antibody (red). *N*^264-39^, *Dl*^*RevF*10 ^and *dAux*^*F*956^* mutant cells are indicated by the absence of nuclear GFP, and the boundaries of mutant clones are outlined with dotted lines. Please note that all *N*^264-39 ^cells are negative for E(spl) staining, whereas some *Dl*^*RevF*10 ^and *dAux*^*F*956^* cells at the clone boundaries are E(spl)-positive (indicated by arrows). Scale bar, 10 μm.

To determine in which cell *dAux *is required during Notch signaling, eye discs containing FLP-induced *dAux*^*F*956^* mutant clones were stained for Enhancer of split (E(spl)), a transcriptional target of Notch signaling [60]. We reasoned that, if *dAux *functions in the receiving cells (cell autonomous), all mutant cells, regardless of their locations within the clones, will be unable to activate Notch and will, therefore, not express E(spl). On the other hand, if *dAux *functions in the signaling cells (non-cell autonomous), mutant cells at the clone border can still receive signals from neighboring wild-type cells, and will express E(spl). Consistent with this reasoning, none of the cells mutant for Notch receptor (*N*^264-39^) within the clones expressed E(spl) (Figure 4A–C) [61,62]. Conversely, as Dl is one of the ligands of this signaling cascade, several *Dl*^*RevF*10 ^mutant cells at the clone border expressed E(spl) (Figure 4D–F) [19]. Similarly, some *dAux*^*F*956^* mutant cells at the clone border clearly expressed E(spl) (Figure 4G–I). This, along with the observations described in previous sections regarding Ato and Cut expression, suggests that, like Dl, *dAux *is required in the signaling cells in the Notch cascade.

### Mutations in *dAux *cause Dl accumulation at the cell surface

**Figure 5:**
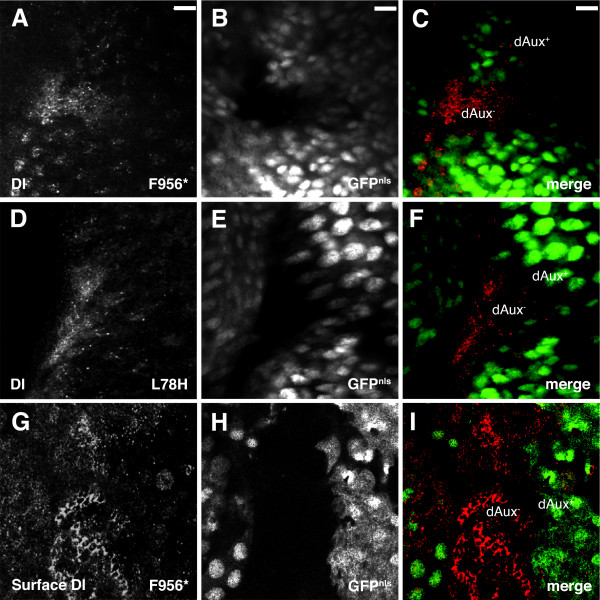
***dAux *mutant cells show an accumulation of Dl at the surface**. Confocal images of (A-C and G-I) *Act5C>FLP/+; FRT*^5-5*Z*3515^, *dAux*^*F*956^*/FRT^5-5*Z*3515^, *ubi-GFP*^*nls *^and (D-F) *Act5C>FLP/+; FRT*^5-5*Z*3515^, *dAux*^*L*78*H*^*/FRT*^5-5*Z*3515^, *ubi-GFP*^*nls *^eye discs stained for Dl (red). In panels A through F, the Dl staining was performed in the presence of detergent. In contrast, the Dl staining in G- I was performed under non-permeabilized conditions, thereby detecting only Dl proteins at the cell surface. *dAux *mutant cells are indicated by the absence of nuclear GFP. Scale bar, 10 μm.

As our genetic data suggest that *dAux *functions in the signaling cells during Notch signaling, a relevant cargo of *dAux*-dependent endocytosis is likely to be Notch ligand. To test this, FLP-induced mutant clones of *dAux*^*L*78*H *^and *dAux*^*F*956^* were first stained with C594.9B α-Dl antibody, which recognizes the extracellular domain of Dl [63]. Compared to the wild-type cells, the intensity of Dl staining was increased in both *dAux*^*F*956^* and *dAux*^*L*78*H *^mutant cells (Figure 5C&F). This increase of Dl staining intensity was more pronounced when the clones were at or near the morphogenetic furrows, consistent with the phenotype shown by a mutation in the PTEN-related region [26].

Although this elevated level of Dl seemed to accumulate around *dAux *mutant cell periphery (Figure 5C&F), it was not clear whether Dl proteins were trapped at the plasma membrane or in vesicular structures inside the cells. To distinguish between these possibilities, eye discs containing *dAux*^*F*956^* mutant clones were stained with C594.9B under a non-permeabilized condition to label Dl at the cell surface. In wild-type tissues, a high level of surface Dl staining was first seen in cells behind the morphogenetic furrow (Figure 5G&I). In more mature clusters located in the posterior region of the eye disc, less Dl was seen at the surface, suggesting that most of Dl was internalized [64]. In *dAux*^*F*956^* mutant clones, the surface Dl staining appeared excessive, indicating that the previously observed peripheral Dl most likely represents Dl accumulated at the cell surface.

### Internalization of EGFR and Notch appear disrupted in *dAux *mutant cells

**Figure 6:**
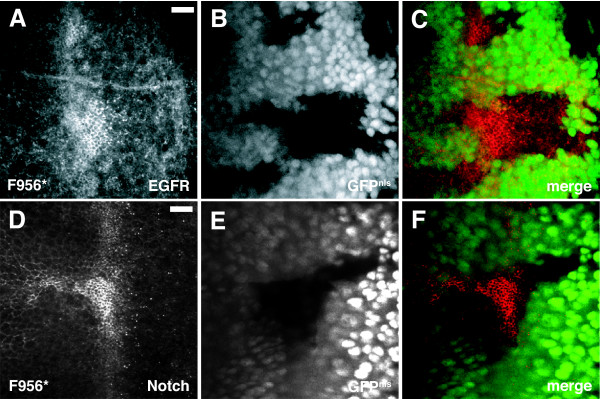
***dAux *mutants show elevated levels of EGF and Notch receptor expression**. Spinning disk confocal images of *Act5C>FLP/+; FRT*^5-5*Z*3515^, *dAux*^*F*956^*/FRT^5-5*Z*3515^, *ubi-GFP*^*nls *^eye disc stained for (A-C) *Drosophila *EGF receptor (red in merged panel) and (D-F) Notch receptor (red in merged panel). *dAux*^*F*956^* mutant cells are indicated by the absence of nuclear GFP. Scale bar, 10 μm.

Knockdown of GAK function in mammalian cells was shown to greatly inhibit the internalization of EGFR [65]. To determine whether the endocytic function of dAux is specific to Notch ligand, FLP-induced *dAux*^*F*956^* mutant eye disc clones were stained with a α-DER antibody [66] and a α-Notch antibody (C17.9C6) [67], respectively. In wild-type eye discs, DER is expressed in cells ahead of furrow, and its expression is reduced behind the furrow [68]. Compared to wild-type cells, DER staining is elevated in the mutant cells (Figure 6A–C), suggesting that the internalization of DER was inhibited. Similarly, the intensity of Notch staining was increased in *dAux*^*F*956^* mutant cells (Figure 6D–F). Thus, although our genetic data suggest that dAux is required in the signaling cells during Notch signaling, the endocytic function of dAux is not limited to the Notch ligand.

### Clathrin distribution was disrupted in dAux mutant clones

**Figure 7:**
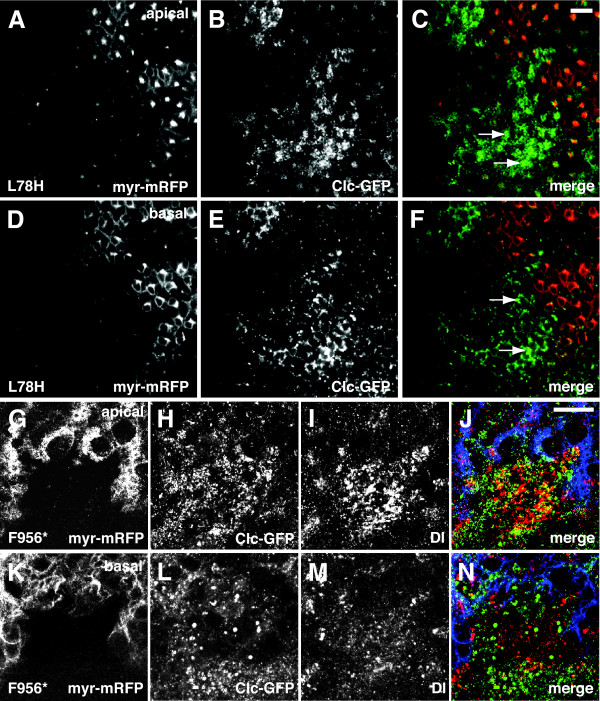
***dAux *mutation disrupts clathrin distribution**. Confocal images of (A-F) *ey>FLP/UAS-EGFP-Clc; FRT*^5-5*Z*3515^, *dAux*^*L*78*H*^*/FRT*^5-5*Z*3515^, *GMR-myr-mRFP *and (G-N) *ey>FLP/UAS-EGFP-Clc; FRT*^5-5*Z*3515^, *dAux*^*F*956^*/FRT^5-5*Z*3515^, *GMR-myr-mRFP *eye discs at two different optical planes (apical: A-C and G-J, and basal: D-F and K-N). For panels A-F, EGFP-tagged Clc, expressed in all cells, is shown in green, and *dAux*^*L*78*H *^mutant cells are indicated by the absence of a membrane-associated mRFP (myr-mRFP, shown in red). For panels G-N, EGFP-Clc is shown in green, Dl staining is shown in red, and *dAux*^*F*956^* mutant cells are indicated by the absence of a membrane-associated myr-mRFP (blue). Arrows indicate the intense Clc-EGFP-positive structures around the cell periphery. The *dAux*^*L*78*H *^clone (A-F) is located in the posterior part of the eye disc, whereas this *dAux*^*F*956^* clone (G-N) is located near the furrow. As a result, the anterior boundary of the *dAux*^*F*956^* clone is not marked because *GMR-myr-mRFP *is only active in cells posterior to the furrow [85]. In the basal section of the *dAux*^*F*956^* clone, less vesicular Dl staining was seen, compared to the nearby wild-type cells. Scale bar, 10 μm.

We have previously observed a genetic interaction between *dAux *and *clathrin light chain *(*Clc*) [26]. To test directly whether clathrin distribution was disrupted by *dAux*, the localization of a Clc-GFP fusion [69] was examined in FLP-induced *dAux *mutant clones in eye discs. In wild-type cells (marked by the presence of a membrane-associated, myr-mRFP), Clc-GFP appeared as vesicular structures near the cell periphery (Figure 7). In both *dAux*^*L*78*H *^and *dAux*^*F*956^* mutant cells (marked by the absence of myr-mRFP), vesicular Clc-GFP still appeared near cell periphery but its intensity was clearly elevated (Figure 7). Furthermore, large and bright spots of Clc-GFP staining could be seen (arrows), suggesting that Clc-GFP or Clc-positive structures may form aggregates in *dAux *mutant cells. These data suggest that normal clathrin distribution depends on both the kinase and the J-domain of dAux. Interestingly, although the level of Dl proteins appeared elevated, staining of Clc-GFP- expressing *dAux*^*F*956^* mutant cells with C594.9B α-Dl antibody showed that Dl was not enriched in these large Clc-positive structures (Figure 7G–N).

### The CBM and J-domains are indispensable for dAux function in Notch signaling

**Figure 8:**
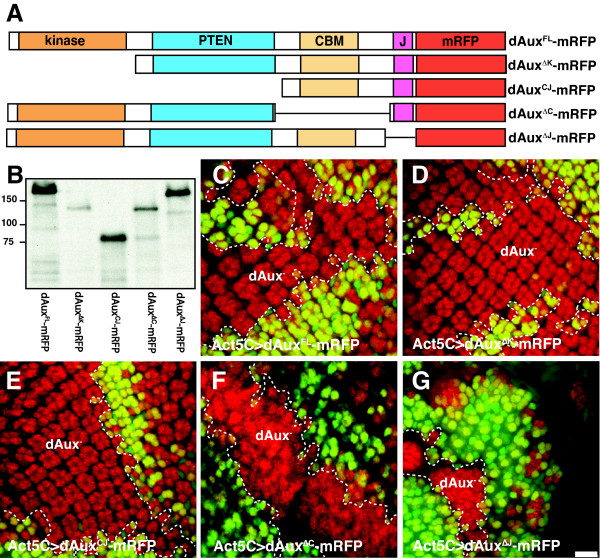
**The CBM and J-domains are necessary for dAux function in Notch signaling**. (A) A schematic diagram depicting various mRFP-tagged dAux constructs (see text for explanation). (B) A Western blot of extracts from flies expressing dAux^FL^-mRFP (lane 1), dAux^ΔK^-mRFP (lane 2), dAux^CJ^-mRFP (lane 3), dAux^ΔC^- mRFP (lane 4), and dAux^ΔJ^-mRFP (lane 5) under the control of *Act5C-Gal4*. The blot was stained with anti-DsRed antibody, which recognizes the mRFP tag. The sizes of protein standards (in kD) are indicated on the left. (C-G) Projected spinning disk confocal images of larval eye discs from (C) *Act5C>FLP/UAS-dAux*^*FL*^*-mRFP; FRT*^5-5*Z*3515^, *dAux*^*F*956^*/FRT^5-5*Z*3515^, *ubi-GFP*^*nls*^, (D) *Act5C>FLP/UAS-dAux*^*ΔK*^*-mRFP; FRT*^5-5*Z*3515^, *dAux*^*F*956^*/FRT^5-5*Z*3515^, *ubi-GFP*^*nls*^, (E) *Act5C>FLP/UAS-dAux*^*CJ*^*-mRFP; FRT*^5-5*Z*3515^, *dAux*^*F*956^*/FRT^5-5*Z*3515^, *ubi-GFP*^*nls*^, (F) *Act5C>FLP/UAS-dAux*^*ΔC*^*-mRFP; FRT*^5-5*Z*3515^, *dAux*^*F*956^*/FRT^5-5*Z*3515^, *ubi-GFP*^*nls*^, and (G) *Act5C>FLP/UAS-dAux*^*ΔJ*^*-mRFP; FRT*^5-5*Z*3515^, *dAux*^*F*956^*/FRT^5-5*Z*3515^, *ubi-GFP*^*nls*^. These eye discs were stained with α-Elav antibody (red), and *dAux*^*F*956^* mutant clones are indicated by the absence of nuclear GFP (green) and outlined by white dotted lines. The fluorescence from mRFP was not shown to present a clearer view of the organization of Elav-positive cells in *dAux *clones. Scale Bar, 10 μm.

To investigate the domains critical for *dAux *function, we generated a series of mRFP-tagged *dAux *derivatives, each with a particular domain or a subset of domains removed (Figure 8A). These include *UAS-dAux*^*FL*^*-mRFP *(full-length), *UAS-dAux*^*ΔK*^*-mRFP *(kinase domain deleted), *UAS-dAux*^*CJ*^*-mRFP *(kinase and PTEN-related domains deleted), *UAS-dAux*^*ΔC*^*-mRFP *(CBM deleted) and *UAS-dAux*^*ΔJ*^*-mRFP *(J-domain deleted). Using a α-DsRed antibody (Clontech) and the *Act5C-GAL4 *driver, bands of expected sizes were detected in blots of extracts prepared from these transgenic flies (Figure 8B), indicating that these truncated dAux proteins were expressed.

To investigate the functional relevance of various dAux domains during Notch signaling, the abilities of these deletions, expressed using the *Act5C-GAL4 *driver, to rescue the supernumerary photoreceptor phenotype exhibited by *dAux *mutant eye discs were determined. While *Act5C-GAL4 *or *UAS-dAux-mRFP *constructs (not shown) alone had no effect, expression of dAux^FL^-mRFP completely suppressed the disorganization and the extra Elav-positive cell defects in FLP-induced *dAux*^*F*956^* clones (Figure 8C). Similarly, expression of dAux^ΔK^-mRFP or dAux^CJ^-mRFP in *dAux*^*F*956^*clones displayed arrays of normal complement of Elav-positive cells, suggesting that over-expression of dAux without its kinase domain and the PTEN-related region can restore normal Notch signaling (Figure 8D&E). In addition, it should be noted that expression of dAux^FL^, dAux^*ΔK *^or dAux^CJ ^could rescue *dAux *mutants to adult viability (data not shown). In contrast, expression of dAux^ΔC^-mRFP and dAux^ΔJ^-mRFP could not rescue the extra Elav-positive cell defects in *dAux*^*F*956^* clones (Figure 8F&G). Over-expression of dAux^ΔJ^-mRFP appeared to have some dominant negative effects. For instance, animals mutant for *dAux*^*L*78*H*^*/dAux*^*W*328^* died during the larval stage. However, these mutants died before the larval stage when the J-domain deletion was expressed under the control of *Act5C-GAL4 *(data not shown). The results of these rescue experiments were confirmed using two independent transgenic lines from each construct and held true for another *dAux *allele (*dAux*^*L*78*H*^), indicating that it is not allele specific. Together, these results suggest that while the kinase and PTEN-related domains are less critical, the J-domain and the CBM region are essential for *dAux *function.

## Discussion

From a F_2 _non-complementation screen, we have isolated several new *dAux *alleles, some of which contain point mutations disrupting specific domains. Consistent with our previous analysis of a viable *dAux *allele, strong *dAux *mutations affect several Notch-mediated processes, including photoreceptor specification in the eye and DV boundary formation in the wing. These phenotypes are consistent with the genetic interactions exhibited between *dAux *and *Notch *[26] and between *dAux *and *lqf *[25]. Taken together, these genetic observations strengthen the notion that endocytosis plays a critical role in Notch signaling, and suggest that *dAux *functions in multiple Notch-dependent events.

As the functional importance of endocytosis has been suggested for both the signaling and receiving cells during Notch signaling [22], it is critical to determine in which cell is dAux function required. Although we have previously concluded that dAux is needed in the signaling cells, the evidence, obtained from mitotic clones of a weak *dAux *allele, was less than convincing [26]. To adequately address this critical issue, we have monitored the expression of E(spl), a Notch target gene, in clones mutant for strong *dAux *alleles. Using these reagents, it is clear that *dAux *mutant cells at the clone border can still activate Notch (a similar result was seen with Cut and Ato staining), suggesting *dAux *acts non-cell autonomously. These genetic data imply that the relevant cargo is likely to be the Notch ligand. Indeed, as shown by the surface labeling experiment, Dl internalization is disrupted in *dAux *mutant cells.

Inhibition of auxilin function by mutations [25,26,42,43], RNAi [31,41,65,70], or injection of inhibitory peptides [71] is known to interfere with the endocytosis of many molecules. In mammalian cells, inhibition of GAK function causes a decrease in the internalization of EGFR and transferrin [31,65]. Our observation suggests that, similar to the mammalian cells, dAux participates in the endocytosis of EGFR, although we did not previously observe a genetic interaction between *DER *and *dAux *[26]. It is possible that this lack of interaction between *dAux *and *DER *reflects the low sensitivity of our genetic assay. Alternatively, it may be that a defect in DER internalization does not significantly impact its signaling during eye development. Consistent with this, we have been unable to detect a drastic increase in the phosphorylation of MAP kinase, a downstream event of DER activation, in *dAux*^*F*956^* mutant clones (data not shown). Nevertheless, our data show that, although the developmental defects of *dAux *resemble those of *Notch*, Notch ligand is not the sole cargo of auxilin-mediated endocytosis. This apparent specificity of dAux's Notch-like phenotypes suggests that the Notch pathway, compared to other signaling cascades, may be more sensitive to disruptions in the clathrin-mediated endocytosis.

Sequencing analysis of our *dAux *alleles revealed that disruptions in the kinase, the PTEN-related region, and the J-domain could all result in abnormal Notch signaling. Noticeably, our screen did not isolate any point mutation in CBM, although the deletion analysis suggests that the CBM is critical for dAux function. This apparent discrepancy is likely due to the fact that the CBM domain contains multiple redundant clathrin-binding motifs [72,73], thereby obscuring the effect of eliminating one single motif by a point mutation. Interestingly, the removal of the CBM from the yeast auxilin (swa-2) does not completely eliminate its function in vivo [73]. The reason for this difference is unclear but it is possible that swa-2 contains other protein domains capable of substituting for the CBM. Similar to a study of the mammalian GAK [31], our deletion analysis confirmed the importance of the J-domain, as over-expression of the dAux^ΔJ ^construct fails to restore the extra photoreceptor cell defect. The CBM and J domains are thought to facilitate the recruitment of Hsc70 to CCVs, and a fragment consisting of CBM and J domain alone has been shown to support clathrin uncoating in vitro [30,33,34]. In support of this notion that the recruitment of Hsc70 to CCVs is likely to be a critical step, over-expression of the CBM and J domain alone could restore the supernumerary Elav-positive cell phenotype.

Conversely, our observation also implies that the loss of the kinase and PTEN-related region could be compensated by the over-expression of the CBM and J-domain. The PTEN-related region is thought to participate in the membrane recruitment of auxilin during CME [35,36]. Thus it is imaginable that a defect in the subcellular localization is less deleterious when the fragment consisting of CBM and J-domain is over-expressed. It is unclear how the requirement of kinase domain can be compensated by the over-expression of the CBM and J-domain, as the relevant substrate for dAux kinase domain during Notch signaling is not known. It should be mentioned that elevated expression of dAux^CJ ^rescued the extra Elav-positive cell phenotype in both *dAux*^*F*956^* and *dAux*^*L*78*H *^(point mutations disrupting the J-domain and the kinase domain respectively), arguing against a scenario in which the kinase domain of endogenous dAux^F956^* mutant proteins could complement the over-expressed dAux^CJ ^in trans. It is possible that some functional redundancy exists between *dAux *and *Numb-associated kinase *(NAK, the *Drosophila *homolog of adaptin-associated kinase) [74], as the kinase domains from both factors are known to phosphorylate adaptor complexes [31,37,75-78]. However, although mutations in subunits of *Drosophila *AP1 and AP2 complexes have been implicated in other Notch-dependent processes [79,80], it is not clear if these adaptor complexes have a role in the Notch processes we examined. Homozygous α *-adaptin *mutants do not appear to exhibit a neurogenic phenotype [69,80]. Furthermore, the removal of one copy of AP2 μ subunit (by a deletion) has no effect on the *dAux*^*I*670*K *^rough eye phenotype (data not shown). In any case, it should be stressed that the kinase and the PTEN-related region do play a role in Notch signaling, as point mutations disrupting these domains cause Notch-like defects, albeit to a weaker extent. Taken together, these results suggest the role of the kinase and the PTEN-related region during Notch ligand endocytosis is less than obligatory.

What is the role of ligand endocytosis in Notch signaling? It has been suggested that, after receptor-ligand binding, ligand endocytosis may provide a mechanical stress or other types of micro-environment (clustered ligand and receptor, etc.) to facilitate Notch cleavage or NECD shedding [21,81]. Alternatively, before binding to Notch, the ligands may have to enter a particular recycling pathway to render them active [20,23,24]. We initially viewed the linking of *dAux *to Notch as evidence favoring the latter model because it suggests that ligand endocytosis needs to proceed past clathrin uncoating. However, as an increased level of the Dl appeared to be trapped at the mutant cell surface, not inside CCVs, the linking of *dAux *to Notch certainly does not exclude the model that ligand internalization per se is critical for Notch signaling. Biochemical analysis has suggested several additional functions for auxilin during the CCV cycle besides uncoating [32]. Although abnormal clathrin distribution was observed in *dAux *cells, given the resolution of our analysis, it is unclear which particular step(s) were affected. It is possible that mutations in *dAux *directly inhibit Notch ligand endocytosis by disrupting one or more of these early steps during CCV formation. Alternatively, *dAux *mutations may indirectly inhibit Notch ligand internalization by causing an excessive formation of non-functional clathrin-dependent structures, thereby decreasing the cytosolic clathrin pool. Indeed, in *dAux *mutant cells, those large clathrin-positive structures did not appear to contain an elevated level of Dl. Consistent with this, it was recently shown that over-expression of Chc could restore the *dAux*-associated defects [27].

## Conclusion

Our genetic analysis of strong *dAux *alleles clearly strengthens the notion that ligand endocytosis plays a critical role in Notch signaling. Furthermore, the deletion analysis suggests that the recruitment of Hsc70 to clathrin is a key event for dAux to facilitate Notch signaling. More importantly, we showed that Dl accumulates at the cell surface in *dAux *mutant cells. This suggests that the linking of *dAux *to the Notch pathway does not exclude the model in which ligand endocytosis activates Notch by physically dissociating the receptor.

## Methods

### Drosophila genetics

All fly crosses were carried out at 25°C in standard laboratory conditions unless otherwise specified. To facilitate the analysis of *dAux *phenotypes in clones, *5-5Z3515*, an *FRT*-containing P-element insertion from the DrosoDel project (Cambridge, UK), was used to isolate additional *dAux *alleles. This strategy was chosen because 1) the conventional *FRT *site for the third chromosome right arm [82] is more distal from the centromere then the *dAux *locus, therefore not applicable for generating *dAux *mutant clones, and 2) crossovers between *FRT*^5-5*Z*3515 ^and *dAux *would have been astronomically rare, as the P element insertion in *5-5Z3515 *is only 20 kb closer to the centromere than the *dAux *locus.

The screens for additional *dAux *alleles on *FRT*^5-5*Z*3515 ^chromosomes were performed as previously described [26]. Briefly, *w; FRT*^5-5*Z*3515 ^males were mutagenized with 25 mM ethyl methane sulfonate (Sigma), and mass mated with *w/w; TM3, Sb/TM6B, Hu *virgins. Progeny were then individually mated with *dAux*^*I*670*K*^, *p [w*^+^*]/TM6B, Hu *flies, and those that failed to complement *dAux*^*I*670*K *^were recovered and maintained over *TM6B *or *TM3 *balancers. To determine the mutations in *dAux *alleles, coding regions were amplified from genomic DNA extracted from homozygous mutant embryos by PCR. Multiple independent PCR products were analyzed by direct sequencing.

### Molecular Biology

The mRFP-tagged dAux deletions were constructed using PCR and standard cloning techniques. The dAux deletions, dAux^ΔK^, dAux^CJ ^and dAux^ΔJ^, correspond to amino acids 339–1165, 762–1165 and 1–998 respectively. dAux^ΔC ^lacks the amino acids 768–1049. In all dAux constructs, mRFP or EGFP was appended in-frame at the C-terminus. The sequence and construction detail will be provided upon request. All the constructs were verified by sequencing, and multiple transgenic flies carrying the constructs were generated by P-element-mediated transformation [83].

### Immunohistochemistry

Immuno-staining of eye and wing imaginal discs was performed as previously described [84]. Rat α-Elav 7E8A10 (DSHB, Iowa), mouse α-Atonal [52], guinea pig α-Senseless [55], mouse α-Cut 2B10 (DSHB, Iowa), α-E(spl) mAB323 [60], mouse α-Dl C594.9B (DSHB, Iowa), mouse α-Notch C17.9C6 (DSHB, Iowa), and rat α-DER [66] were used at 1:100, 1:3000, 1:1000, 1:100, 1:2, 1:100, 1:100, and 1:50 dilutions, respectively. Fluorescently conjugated secondary antibodies (Molecular Probes) were used according to the manufacturer's instructions. Fluorescent microscopy was performed using the Olympus BX61 microscope equipped with the Olympus DSU confocal system and processed with Photoshop (Adobe) and Volocity (Improvision).

For surface Dl labeling, mosaic eye discs were dissected and fixed in 4% paraformaldehyde. The peripodial membranes were partially removed, and the discs were then stained with mouse α-Dl C594.9B and washed with PBS in the absence of any detergent. The stained eye discs were examined using a BioRad MRC1024 laser confocal microscope (Nikon OPTIPHOT-2) and the images were processed with Adobe Photoshop.

### Western analysis and fly extract preparation

To prepare fly extracts, flies expressing mRFP-tagged dAux derivatives were homogenized in 2× SDS-loading buffer containing 0.2 mM DTT (10 flies/100μl), boiled immediately for 5 minutes and separated by SDS-PAGE using 10% acrylamide gel. The gel was transferred to nitrocellulose membrane and probed with α-DsRed rabbit polyclonal antibody (Clontech) at 1:1000 dilution, followed by HRP-conjugated goat anti-rabbit secondary antibody (Jackson Lab). The immunodetection was performed using ECL substrate (Amersham Biosciences).

## Authors' contributions

TB and VK carried out the isolation and molecular characterization of the *dAux *alleles. VK performed the phenotypic analysis of the *dAux *mutants and the deletion analysis of dAux domains. HCC was responsible for most of the experimental design and the manuscript preparation.
